# Surgical Approach and Long-Term Recurrence After Ventral Hernia Repair

**DOI:** 10.1001/jamasurg.2024.1696

**Published:** 2024-06-12

**Authors:** Brian T. Fry, Ryan A. Howard, Jyothi R. Thumma, Edward C. Norton, Justin B. Dimick, Kyle H. Sheetz

**Affiliations:** 1 Department of Surgery, University of Michigan, Ann Arbor; 2 Center for Healthcare Outcomes & Policy, University of Michigan, Ann Arbor; 3 Department of Health Management and Policy, University of Michigan, Ann Arbor; 4 Department of Economics, University of Michigan, Ann Arbor; 5 Surgical Innovation Editor, *JAMA Surgery*

## Abstract

**Importance:**

The prevalence of robotic-assisted anterior abdominal wall (ventral) hernia repair has increased dramatically in recent years, despite conflicting evidence of patient benefit. Whether long-term hernia recurrence rates following robotic-assisted repairs are lower than rates following more established laparoscopic or open approaches remains unclear.

**Objective:**

To evaluate the association between robotic-assisted, laparoscopic, and open approaches to ventral hernia repair and long-term operative hernia recurrence.

**Design, Setting, and Participants:**

Secondary retrospective cohort analysis using Medicare claims data examining adults 18 years and older who underwent elective inpatient ventral, incisional, or umbilical hernia repair from January 1, 2010, to December 31, 2020. Data analysis was performed from January 2023 through March 2024.

**Exposure:**

Operative approach to ventral hernia repair, which included robotic-assisted, laparoscopic, and open approaches.

**Main Outcomes and Measures:**

The primary outcome was operative hernia recurrence for up to 10 years after initial hernia repair. To help account for potential bias from unmeasured patient factors (eg, hernia size), an instrumental variable analysis was performed using regional variation in the adoption of robotic-assisted hernia repair over time as the instrument. Cox proportional hazards modeling was used to estimate the risk-adjusted cumulative incidence of operative recurrence up to 10 years after the initial procedure, controlling for factors such as patient age, sex, race and ethnicity, comorbidities, and hernia subtype (ventral/incisional or umbilical).

**Results:**

A total of 161 415 patients were included in the study; mean (SD) patient age was 69 (10.8) years and 67 592 patients (41.9%) were male. From 2010 to 2020, the proportion of robotic-assisted procedures increased from 2.1% (415 of 20 184) to 21.9% (1737 of 7945), while the proportion of laparoscopic procedures decreased from 23.8% (4799 of 20 184) to 11.9% (946 of 7945) and of open procedures decreased from 74.2% (14 970 of 20 184) to 66.2% (5262 of 7945). Patients undergoing robotic-assisted hernia repair had a higher 10-year risk-adjusted cumulative incidence of operative recurrence (13.43%; 95% CI, 13.36%-13.50%) compared with both laparoscopic (12.33%; 95% CI, 12.30%-12.37%; HR, 0.78; 95% CI, 0.62-0.94) and open (12.74%; 95% CI, 12.71%-12.78%; HR, 0.81; 95% CI, 0.64-0.97) approaches. These trends were directionally consistent regardless of surgeon procedure volume.

**Conclusions and Relevance:**

This study found that the rate of long-term operative recurrence was higher for patients undergoing robotic-assisted ventral hernia repair compared with laparoscopic and open approaches. This suggests that narrowing clinical applications and evaluating the specific advantages and disadvantages of each approach may improve patient outcomes following ventral hernia repairs.

## Key Points

**Question:**

How do long-term operative recurrence rates for anterior abdominal wall (ventral) hernia compare after robotic-assisted, laparoscopic, and open hernia repairs?

**Findings:**

This cohort study of Medicare claims data identified 161 415 patients who underwent ventral hernia repair from January 2010 to December 2020. Up to 10 years after surgery, patients who underwent robotic-assisted hernia repair had higher rates of operative recurrence (13.4%) than patients who underwent laparoscopic (12.3%) or open (12.7%) repair.

**Meaning:**

Higher long-term recurrence rates after robotic-assisted ventral hernia repair compared with other methods call into question the rapid and widespread adoption of this approach over the last decade.

## Teaser

This cohort study uses Medicare claims data to analyze the operative recurrence rate for ventral hernia for up to 10 years after patients underwent robotic-assisted, laparoscopic, and open ventral hernia repairs from January 2010 to December 2020.

## Introduction

Robotic-assisted surgery is rapidly expanding into new clinical domains.^1^ This is especially true for anterior abdominal wall (ventral) hernia repair, where use of robotic-assisted repair increased 45-fold between 2012 and 2018 alone.^2,3^ When compared with an open procedure, proponents of robotic-assisted hernia repair cite the benefits of a minimally invasive approach, such as smaller incisions and fewer postoperative complications. They also cite specific advantages over laparoscopy, including an easier learning curve, improved dexterity, 3-dimensional visualization (compared with 2-dimensional laparoscopy), and better ergonomics for surgeon longevity.^4,5,6,7^

Despite the rapid adoption of robotic-assisted ventral hernia repair, it remains unclear whether long-term outcomes, such as hernia recurrence, are superior for a robotic-assisted approach compared with more established laparoscopic or open approaches. Existing observational studies and randomized trials comparing robotic-assisted, laparoscopic, and open ventral hernia repair are limited in several important ways. First, most observational studies are conducted at a single institution with small sample sizes, or focus only on short-term outcomes, such as 30-day complications or length of stay.^8,9,10,11,12,13,14,15^ Those studies that include longer-term outcomes, such as hernia recurrence, are limited by incomplete or relatively short follow-up durations.^16,17,18,19^ Moreover, existing population-level studies demonstrate conflicting data on recurrence after robotic-assisted repair and do not account for selection bias, limiting researchers’ ability to infer causality between operative approach and recurrence.^20,21^ Finally, existing randomized clinical trials include only high-volume, experienced robotic-assisted surgeons and do not accurately reflect the heterogenous training and expertise of most surgeons performing hernia repairs across the country.^16,17,18^

We sought to evaluate long-term recurrence following robotic-assisted, laparoscopic, and open ventral hernia repair among a national cohort of Medicare beneficiaries. This allowed us to evaluate real-world outcomes using population-level data with longitudinal follow-up, due to near-universal enrollment and low disenrollment rates. Using Medicare data also allowed us to leverage regional differences in the adoption of robotic-assisted ventral hernia repair as an instrumental variable (IV) to account for confounding from unmeasured factors that may bias comparisons. A better understanding of the comparative outcomes for each approach can inform the evidence-based adoption of robotic-assisted ventral hernia repair and further our knowledge of the true benefits of this technology.

## Methods

### Data Source and Patient Population

We used data from the Medicare Provider Analysis and Review Parts A and B files to identify initial (index) operations for patients 18 years or older who underwent elective, inpatient ventral hernia repair from January 1, 2010, through December 31, 2020. Ventral hernia included all anterior abdominal wall hernias coded in claims as ventral, incisional, umbilical, or epigastric hernia repair. Patients were identified initially using *International Classification of Diseases, Ninth Revision *(*ICD-9*) and *ICD-10* procedure codes, which were cross-referenced with the corresponding *ICD-9* and *ICD-10* diagnosis codes (eTable 1 in Supplement 1). Patients were excluded from the index cohort if they had a prior hernia repair in the 2 years leading up to the initial operation or if the operation was associated with *Current Procedural Terminology* (*CPT*) codes for repair of a recurrent ventral hernia (codes 49565, 49566, 49656, or 49657). All subsequent admissions for a ventral hernia operation after the index repair were excluded from the index cohort. Patients not enrolled in fee-for-service Medicare were also excluded due to lack of accurate follow-up. To ensure we accurately captured index hernia repairs from *ICD-9* and *ICD-10* procedure codes, patients were also excluded if they lacked a concurrent *CPT* code for hernia repair (eTable 1 in Supplement 1). A flow diagram with detailed stepwise patient inclusion and exclusion is available in eFigure 1 in Supplement 1. This study used deidentified patient data and was therefore deemed exempt from regulation by the University of Michigan Institutional Review Board. This study followed the Strengthening the Reporting of Observational Studies in Epidemiology (STROBE) reporting guidelines.

### Outcome Measures and Explanatory Variables

The primary outcome was operative recurrence for ventral hernia repair. Operative recurrence was used as a proxy for true clinical recurrence, which cannot be measured in Medicare claims data alone. The total number of ventral/incisional and umbilical hernia repairs was tabulated for each year studied. Surgical approach (robotic-assisted, laparoscopic, or open) was identified using corresponding *CPT*, *ICD-9*, and *ICD-10* codes (eTable 1 in Supplement 1). Operation for recurrence was identified by a subsequent hernia repair using the same *CPT*, *ICD-9*, and *ICD-10* codes used to identify the initial hernia repair, the presence of specific hernia recurrence *CPT* codes (codes 49565, 49566, 49656, and 49657), or both methods.

Explanatory variables included in our models were consistent with prior published work on hernia and included patient age, sex, race and ethnicity (eMethods 1 in Supplement 1), Elixhauser comorbidities, year of surgery, approach (robotic-assisted, laparoscopic, or open), mesh use, the use of myofascial flap, and hernia subtype (ventral/incisional or umbilical).^20,21^ Patient race and ethnicity are included as identifiers in the Medicare claims database in this study and are represented in the following standard, fixed categories: Asian, Black, Hispanic, North American Native, White, other race, and unknown race. Only a single category could be selected, and no additional information is available in Medicare claims data regarding the composition of the other race category. Age was treated as a continuous variable. All other variables were treated as categorical.

### IV Analysis

The rapid uptake of robotic-assisted hernia repair necessitates the use of existing observational data, as opposed to launching a large-scale randomized clinical trial, to assess long-term outcomes after robotic-assisted, laparoscopic, and open ventral hernia repair. However, due to measured and unmeasured confounding in observational data, causal inference may be limited by selection bias. Standard multivariable logistic regression analysis would not account for this selection bias, as it can only adjust for measured covariates. One known strategy to mitigate this bias is to perform an IV analysis. For additional information, including the reasoning behind and justification for the choice of an IV analysis, please see eMethods 2 in Supplement 1.

The IV used for this analysis was the rate of use of robotic-assisted ventral hernia repair within a hospital referral region (HRR) in the 12 months prior to a patient’s initial ventral hernia repair. Using prior-year state-level and regional-level procedural use as an IV is consistent with previous observational studies in surgical patients.^22,23^ We excluded patients located in HRRs with no robotic-assisted hernia repairs during the study period.

### Statistical Analysis

Due to the binary, nonlinear outcome of operative hernia recurrence, time-to-event analysis was performed using a 2-stage residual inclusion estimation method for our IV model.^24^ For the first stage, multivariable logistic regression was used to estimate the likelihood that a patient would undergo robotic-assisted ventral hernia repair, while adjusting for the following covariates: prior-year HRR use of robotic-assisted ventral hernia repair (the IV), age, sex, race and ethnicity, comorbidities, myofascial flap use, and hernia subtype. Mesh use was excluded from this first stage, as it was perfectly collinear with robotic-assisted use. For the second stage, a Cox proportional hazards model was constructed to calculate hazard ratios (HRs) and the cumulative incidence of operative hernia recurrence, while adjusting for the following covariates: surgical approach, age, sex, race and ethnicity, comorbidities, hernia subtype, myofascial flap use, mesh use, year of surgery, and residuals from the first-stage regression model, which represent unmeasured confounding associated with the choice of operative approach. The second stage of this model also accounted for clustering of outcomes at the HRR level. Patients were censored if they died, disenrolled from Medicare, or reached the end of the study period. Hazard ratios were calculated using marginal effect estimates from the Cox models. Operative recurrence numbers were estimated using the Cox models with covariates set to their mean values. Proportional hazards assumptions were tested using Schoenfeld residuals. For variables that violated this assumption, we included an interaction term with the logarithm of time in the second stage of our model.^25^ Variables that required an interaction term with time included surgical approach, age, sex, chronic pulmonary disease, hypothyroidism, kidney failure, fluid and electrolyte disorders, and obesity.

To assess the strength of the IV, we first calculated the Kleibergen-Paap Wald *F* statistic for prior-year use of robotic-assisted ventral hernia repair and the current-year treatment (eg, undergoing robotic-assisted ventral hernia repair). Our *F* statistic of 63 966 demonstrated that our IV was highly associated with undergoing robotic-assisted ventral hernia, as an *F* statistic greater than 10 is generally considered to be a strong instrument.^26^ A valid instrument must not be associated with the outcome except through the treatment variable. While this condition cannot be empirically proven, it can be evaluated on both a theoretical basis and by examining the balance of patient characteristics when stratified by the instrument. For the former theoretical reasoning, local lagged treatment patterns are believed to satisfy this condition, as they reflect clinician treatment decisions from a prior time period among a different set of patients.^27^ Additionally, regional use of robotic-assisted surgery in a previous year is highly likely to influence its use in the subsequent year. For the latter stratification, we analyzed baseline patient characteristics for as-treated cohorts and for patients stratified around the median of our IV (eTables 2 and 3 in Supplement 1). With a strong instrument, patient-level covariates are ideally more similar across approaches when comparing the above-median and below-median levels of the instrument to the actual treatment level.

There was a significant reduction in covariate imbalance when evaluating by above-median and below-median groups of the IV vs the as-treated operative approach (eTables 2 and 3 in Supplement 1). For example, a standardized difference of 8.3% in the prevalence of chronic pulmonary disease between actual treatment groups (robotic-assisted: 2683 [21.1%]; laparoscopic: 8014 [24.6%]) was reduced to 0.1% using the instrument (robotic-assisted: 18 841 [23.4%]; laparoscopic: 18 999 [23.5%]). After implementation of the instrument, the number of covariates with a standardized difference greater than 10% was reduced compared to the as-treated covariates for robotic-assisted vs laparoscopic and robotic-assisted vs open approaches. Thus, we used an IV for our main analysis.

Several preplanned sensitivity analyses were performed in an identical manner restricting the patient population by hernia subtype (either ventral/incisional or umbilical), presence of obesity, number of comorbidities, *ICD-9* and *ICD-10* coding status, surgeon volume of robotic-assisted ventral hernia repairs, and the proportion of an individual surgeon’s ventral hernia cases performed with robotic assistance. An additional sensitivity analysis was performed post hoc looking at myofascial flap use. Instrumental variable models were also run with clustering at either the hospital or surgeon level; however, this did not appreciably affect the CIs of our estimates. All analyses were performed using SAS version 9.4 (SAS Institute) and Stata version 15 (StataCorp LLC). Tests were 2-sided and significance was set at *P* < .05. Baseline unadjusted patient characteristics were compared across the 3 approaches using analysis of variance, Kruskal Wallis, or Pearson χ^2^ tests as appropriate. The IV analysis used robust standard errors to account for HRR-level heteroscedasticity. Analysis was performed from January 2023 through March 2024.

## Results

### Patient Characteristics

From 2010 to 2020, 161 415 patients underwent ventral hernia repair and were included in our study (eFigure 1 in Supplement 1), 12 693 procedures of which were robotic assisted, 32 542 were laparoscopic, and 116 180 were open (Table 1). The mean (SD) patient age was 69.0 (10.8) years and 67 592 patients (41.9%) were male. The total number of elective inpatient ventral hernia repairs decreased each year, from 20 184 in 2010 to 7945 in 2020. During this time, the annual number and proportion of robotic-assisted repairs increased from 415 of 20 184 total procedures (2.1%) in 2010 to 1737 of 7945 total procedures (21.9%) in 2020; laparoscopic repairs decreased from 4799 (23.8%) to 946 (11.9%); and open repairs decreased from 14 970 (74.2%) to 5262 (66.2%) (eFigure 2 in Supplement 1). The median (IQR) follow-up time after initial hernia repair was 64.0 (24.9-101.5) months. A total of 15 815 patients (9.8%) had full follow-up data available at 10 years postoperatively.

**Table 1.** Cohort Characteristics by Operative Approach

Characteristic	Operative approach, No. (%)				*P* value^a^
	All	Robotic-assisted	Laparoscopic	Open	
Total	161 415 (100)	12 693 (7.8)	32 542 (20.2)	116 180 (72.0)	NA
Age, mean (SD), y	69.0 (10.8)	69.9 (8.9)	68.5 (11.1)	69.0 (10.9)	<.001
Sex					
Female	93 823 (58.1)	5810 (45.8)	19 992 (61.4)	68 021 (58.5)	<.001
Male	67 592 (41.9)	6883 (54.2)	12 550 (38.6)	48 159 (41.5)	
Race and ethnicity					
Asian	871 (0.5)	89 (0.7)	152 (0.5)	630 (0.5)	.009
Black	13 885 (8.6)	1048 (8.3)	2707 (8.3)	10 130 (8.7)	.03
Hispanic	3155 (2.0)	227 (1.8)	593 (1.8)	2335 (2.0)	.04
White	139 179 (86.2)	10 911 (86.0)	28 285 (86.9)	99 983 (86.1)	<.001
Comorbidities^b^					
Hypertension	108 223 (67.0)	8640 (68.1)	22 072 (67.8)	77 511 (66.7)	<.001
Obesity	37 920 (23.5)	3281 (25.8)	7606 (23.4)	27 033 (23.3)	<.001
Chronic pulmonary disease	37 840 (23.4)	2683 (21.1)	8014 (24.6)	27 143 (23.4)	<.001
Diabetes without chronic complications	36 631 (22.7)	2415 (19.0)	8077 (24.8)	26 139 (22.5)	<.001
Hypothyroidism	24 991 (15.5)	1861 (14.7)	5215 (16.0)	17 915 (15.4)	.001
Fluid and electrolyte disorders	24 274 (15.0)	1565 (12.3)	3571 (11.0)	19 138 (16.5)	<.001
Depression	19 516 (12.1)	1400 (11.0)	4270 (13.1)	13 846 (11.9)	<.001
Kidney failure	18 712 (11.6)	1405 (11.1)	3304 (10.2)	14 003 (12.1)	<.001
Deficiency anemias	18 339 (11.4)	1211 (9.5)	2915 (9.0)	14 213 (12.2)	<.001
Congestive heart failure	12 655 (7.8)	920 (7.2)	2372 (7.3)	9363 (8.1)	<.001
Diabetes with chronic complications	9786 (6.1)	1076 (8.5)	1695 (5.2)	7015 (6.0)	<.001
Peripheral vascular disease	8300 (5.1)	511 (4.0)	1577 (4.8)	6212 (5.3)	<.001
Other neurological disorders	7777 (4.8)	492 (3.9)	1523 (4.7)	5762 (5.0)	<.001
Valvular disease	7487 (4.6)	556 (4.4)	1522 (4.7)	5409 (4.7)	.35
Liver disease	7163 (4.4)	428 (3.4)	1563 (4.8)	5172 (4.5)	<.001
Weight loss	5155 (3.2)	263 (2.1)	535 (1.6)	4357 (3.8)	<.001
Rheumatoid arthritis or collagen vascular disease	5104 (3.2)	354 (2.8)	1017 (3.1)	3733 (3.2)	.03
Psychoses	4578 (2.8)	262 (2.1)	907 (2.8)	3409 (2.9)	<.001
Coagulopathy	4313 (2.7)	299 (2.4)	629 (1.9)	3385 (2.9)	<.001
Metastatic cancer	4296 (2.7)	300 (2.4)	344 (1.1)	3652 (3.1)	<.001
Solid tumor without metastasis	3366 (2.1)	267 (2.1)	470 (1.4)	2629 (2.3)	<.001
Hernia type					
Ventral or incisional	131 021 (81.2)	7674 (60.5)	28 426 (87.4)	94 921 (81.7)	<.001
Umbilical	30 394 (18.8)	5019 (39.5)	4116 (12.6)	21 259 (18.3)	<.001
Hernia technique					
Mesh use^c^	109 907 (68.1)	12 693 (100)	32 542 (100)	64 672 (55.7)	<.001
Myofascial flap	20 625 (12.8)	818 (6.4)	597 (1.8)	19 210 (16.5)	<.001
Year of surgery					
2010	20 184 (12.5)	415 (3.3)	4799 (14.7)	14 970 (12.9)	<.001
2011	19 465 (12.1)	516 (4.1)	4535 (13.9)	14 414 (12.4)	
2012	18 153 (11.2)	574 (4.5)	4259 (13.1)	13 320 (11.5)	
2013	16 706 (10.3)	626 (4.9)	3848 (11.8)	12 232 (10.5)	
2014	15 145 (9.4)	791 (6.2)	3275 (10.1)	11 079 (9.5)	
2015	14 247 (8.8)	1032 (8.1)	2806 (8.6)	10 409 (9.0)	
2016	13 888 (8.6)	1453 (11.4)	2574 (7.9)	9861 (8.5)	
2017	13 247 (8.2)	1763 (13.9)	2197 (6.8)	9287 (8.0)	
2018	11 618 (7.2)	1826 (14.4)	1781 (5.5)	8011 (6.9)	
2019	10 817 (6.7)	1960 (15.4)	1522 (4.7)	7335 (6.3)	
2020	7945 (4.9)	1737 (13.7)	946 (2.9)	5262 (4.5)	

Abbreviation: NA, not applicable.

^a^ *P* values shown are for analysis of variance, Kruskal-Wallis, or Pearson χ^2^ tests depending on variable type.

^b^ Comorbidities with a sample prevalence of at least 2% reported.

^c^ Mesh use includes any open procedure with a separate billing claim for mesh placement or any minimally invasive procedure (robotic, laparoscopic), as mesh placement is included in the billing claim for these case.

**Figure 1.**
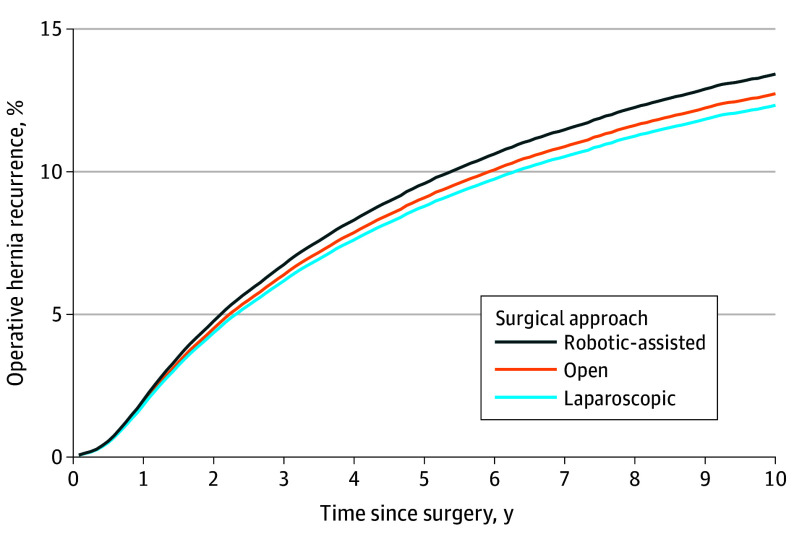
Overall Cumulative Incidence of Operative Recurrence Following Ventral Hernia Repair, Stratified by Approach (Robotic, Laparoscopic, and Open) From 2010-2020 Cumulative incidence of operative hernia recurrence was calculated using a Cox proportional hazards model that adjusted for patient age, sex, race and ethnicity, Elixhauser comorbidities, year of surgery, approach (robotic-assisted, laparoscopic, or open), mesh use, the use of myofascial flap, and procedure type (ventral/incisional or umbilical). Analysis included use of an instrumental variable to reduce measured and unmeasured confounding. The instrument used was robotic-assisted ventral hernia repair use rate within a hospital referral region in the 12 months prior to a patient’s initial ventral hernia repair. 95% CIs are not visible as the largest interval was −0.18% to 0.18% from point estimates.

**Figure 2.**
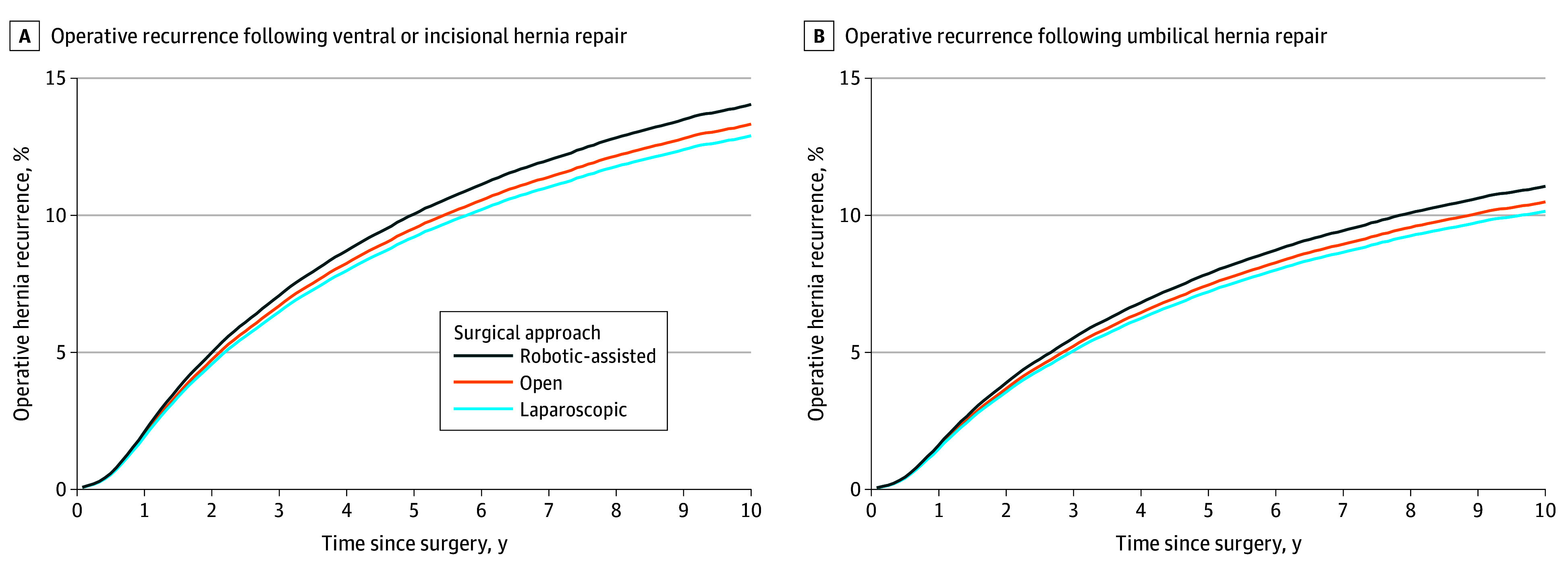
Cumulative Incidence of Operative Recurrence Following Ventral or Incisional Subtype Hernia Repairs Only and Umbilical Hernia Subtype Repairs Only, Stratified by Approach (Robotic-Assisted, Laparoscopic, and Open) From 2010-2020 Cumulative incidence of operative hernia recurrence following ventral or incisional (A) and umbilical (B) hernia repair was calculated using a Cox proportional hazards model that adjusted for patient age, sex, race and ethnicity, Elixhauser comorbidities, year of surgery, approach (robotic-assisted, laparoscopic, or open), mesh use, the use of myofascial flap, and hernia subtype (ventral/incisional or umbilical). Analysis included use of an instrumental variable to reduce measured and unmeasured confounding. The instrument used was robotic-assisted ventral hernia repair use rate within a hospital referral region in the 12 months prior to a patient’s initial ventral hernia repair. 95% CIs are not visible as the largest interval was −0.18% to 0.18% from point estimates for ventral and incisional subtype hernias and −0.14% to 0.14% from point estimates for umbilical-subtype hernias.

Throughout the entire study period, unadjusted operative recurrence rates were 10.3% after robotic-assisted repair (1302 recurrences following 12 693 repairs), 14.0% after laparoscopic repair (4559 recurrences following 32 542 repairs), and 13.4% after open ventral hernia repair (15 566 recurrences following 116 180 repairs). After risk adjustment and running our 2-stage IV Cox proportional hazards model, patients who underwent robotic-assisted ventral hernia repair had a higher cumulative incidence of operative hernia recurrence up to 10 years after surgery (13.43%; 95% CI, 13.36%-13.50%) compared to patients who underwent laparoscopic repair (12.33%; 95% CI, 12.30%-12.37%) and open repair (12.74%; 95% CI, 12.71%-12.78%) (Figure 1 and Table 2). This corresponds to an adjusted HR of reoperation following laparoscopic hernia repair of 0.78 (95% CI, 0.62-0.94) and an HR of 0.81 (95% CI, 0.64-0.97) following open hernia repair compared with robotic-assisted hernia repair (Table 2). These trends were similar when stratified by hernia subtypes of ventral/incisional and umbilical (Figure 2).

**Table 2.** Risk-Adjusted Cumulative Incidence of Operative Recurrence and Risk-Adjusted Hazard Ratios at 1, 3, 5, 7, and 10 Years After Robotic-Assisted, Laparoscopic, and Open Ventral Hernia Repair From 2010-2020, Stratified by Hernia Subtype

Hernia repair type	Years after initial repair									
	Cumulative incidence rate (95% CI)					Hazard ratio (95% CI)				
	1	3	5	7	10	1	3	5	7	10
**All ventral hernias**										
Robotic-assisted	2.00 (1.98-2.00)	6.75 (6.72-6.78)	9.59 (9.55-9.63)	11.48 (11.43-11.53)	13.43 (13.36-13.50)	1 [Reference]	1 [Reference]	1 [Reference]	1 [Reference]	1 [Reference]
Laparoscopic	1.82 (1.82-1.83)	6.18 (6.17-6.19)	8.79 (8.78-8.81)	10.53 (10.51-10.56)	12.33 (12.30-12.37)	0.95 (0.82-1.08)	0.86 (0.74-0.99)	0.83 (0.69-0.97)	0.80 (0.66-0.95)	0.78 (0.62-0.94)
Open	1.89 (1.88-1.90)	6.39 (6.38-6.40)	9.09 (9.08-9.10)	10.89 (10.87-10.91)	12.74 (12.71-12.78)	0.97 (0.85-1.10)	0.89 (0.76-1.02)	0.85 (0.71-1.00)	0.83 (0.68-0.98)	0.81 (0.64-0.97)
**Umbilical hernia subtype only**										
Robotic-assisted	1.63 (1.62-1.63)	5.52 (5.50-5.55)	7.87 (7.84-7.90)	9.44 (9.40-9.48)	11.07 (11.01-11.13)	1 [Reference]	1 [Reference]	1 [Reference]	1 [Reference]	1 [Reference]
Laparoscopic	1.49 (1.48-1.49)	5.06 (5.05-5.06)	7.21 (7.20-7.23)	8.66 (8.64-8.67)	10.15 (10.12-10.19)	0.96 (0.81-1.10)	0.87 (0.73-1.01)	0.83 (0.68-0.98)	0.81 (0.65-0.97)	0.78 (0.61-0.95)
Open	1.54 (1.53-1.54)	5.23 (5.22-5.24)	7.46 (7.44-7.47)	8.95 (8.93-8.97)	10.49 (10.46-10.52)	0.94 (0.83-1.05)	0.86 (0.75-0.98)	0.83 (0.70-0.96)	0.81 (0.67-0.94)	0.78 (0.63-0.93)
**Ventral or incisional subtype hernia only**										
Robotic-assisted	2.09 (2.08-2.10)	7.07 (7.04-7.10)	10.04 (10.00-10.08)	12.02 (11.96-12.07)	14.05 (13.97-14.13)	1 [Reference]	1 [Reference]	1 [Reference]	1 [Reference]	1 [Reference]
Laparoscopic	1.91 (1.91-1.92)	6.48 (6.47-6.49)	9.21 (9.19-9.23)	11.03 (11.01-11.05)	12.91 (12.87-12.95)	0.82 (0.74-0.89)	0.74 (0.67-0.82)	0.71 (0.62-0.80)	0.69 (0.59-0.79)	0.67 (0.56-0.78)
Open	1.98 (1.98-1.98)	6.70 (6.69-6.71)	9.52 (9.51-9.53)	11.40 (11.38-11.42)	13.33 (13.29-13.37)	0.87 (0.80-0.95)	0.80 (0.72-0.88)	0.77 (0.67-0.87)	0.75 (0.64-0.86)	0.73 (0.61-0.85)

### Sensitivity Analyses

We performed multiple sensitivity analyses to test our results (Table 3). The previously mentioned findings were consistent across patients with obesity vs patients without obesity, patients with a low vs high comorbidity burden, when looking at *ICD-9* vs *ICD-10* coding, and for those without myofascial flap repair. For those hernia repairs that included myofascial flaps, open repair had lower 10-year operative recurrence rates while laparoscopic repair had higher recurrence rates than robotic-assisted repair. Robotic-assisted repair was associated with higher operative recurrence rates compared with the other 2 approaches across varying levels of surgeon robotic-assisted ventral hernia repair volume, although the magnitude of these differences was smaller for surgeons with the highest proportion of robotic-assisted ventral hernia repairs (ie, in the top 25%).

**Table 3.** Sensitivity Analyses With Hazard Ratios (HRs) for Operative Recurrence at 1, 3, 5, 7, and 10 Years After Robotic-Assisted, Laparoscopic, and Open Ventral Hernia Repair From 2010-2020

Variable	Years after repair, HR (95% CI)				
	1	3	5	7	10
**Obesity**					
Patients with obesity					
Robotic-assisted	1 [Reference]	1 [Reference]	1 [Reference]	1 [Reference]	1 [Reference]
Laparoscopic	0.84 (0.74-0.94)	0.77 (0.67-0.87)	0.74 (0.63-0.85)	0.72 (0.60-0.84)	0.70 (0.57-0.83)
Open	0.92 (0.82-1.02)	0.85 (0.75-0.95)	0.82 (0.70-0.93)	0.79 (0.67-0.92)	0.77 (0.64-0.91)
Patients without obesity					
Robotic-assisted	1 [Reference]	1 [Reference]	1 [Reference]	1 [Reference]	1 [Reference]
Laparoscopic	0.95 (0.87-1.03)	0.87 (0.77-0.96)	0.83 (0.72-0.94)	0.81 (0.69-0.93)	0.79 (0.65-0.93)
Open	0.95 (0.90-1.05)	0.90 (0.80-0.99)	0.86 (0.75-0.98)	0.84 (0.72-0.97)	0.82 (0.68-0.96)
**No. of comorbidities**					
0-1					
Robotic-assisted	1 [Reference]	1 [Reference]	1 [Reference]	1 [Reference]	1 [Reference]
Laparoscopic	0.95 (0.85-1.07)	0.88 (0.77-0.99)	0.84 (0.72-0.96)	0.82 (0.68-0.95)	0.79 (0.65-0.94)
Open	0.97 (0.82-1.14)	0.93 (0.83-1.04)	0.90 (0.77-1.02)	0.87 (0.74-1.01)	0.85 (0.70-1.00)
≥2					
Robotic-assisted	1 [Reference]	1 [Reference]	1 [Reference]	1 [Reference]	1 [Reference]
Laparoscopic	0.91 (0.83-0.98)	0.83 (0.74-0.91)	0.79 (0.69-0.90)	0.77 (0.66-0.89)	0.75 (0.62-0.88)
Open	0.94 (0.87-1.00)	0.86 (0.77-0.94)	0.82 (0.72-0.93)	0.80 (0.68-0.92)	0.78 (0.65-0.91)
**Myofascial flap^a^**					
Flap use					
Robotic-assisted	1 [Reference]	1 [Reference]	1 [Reference]	1 [Reference]	1 [Reference]
Laparoscopic	2.74 (1.88-3.6)	2.43 (1.65-3.20)	2.30 (1.53-3.06)	2.21 (1.45-2.98)	2.13 (1.36-2.90)
Open	0.49 (0.36-0.63)	0.43 (0.31-0.55)	0.40 (0.28-0.52)	0.39 (0.27-0.51)	0.37 (0.25-0.49)
No flap use					
Robotic-assisted	1 [Reference]	1 [Reference]	1 [Reference]	1 [Reference]	1 [Reference]
Laparoscopic	0.74 (0.66-0.81)	0.65 (0.57-0.73)	0.62 (0.52-0.71)	0.59 (0.49-0.70)	0.57 (0.45-0.69)
Open	0.89 (0.80-0.97)	0.77 (0.68-0.86)	0.72 (0.61-0.84)	0.69 (0.57-0.82)	0.66 (0.52-0.80)
***ICD* version**					
*ICD-9* (2010-September 2015)^b^					
Robotic-assisted	1 [Reference]	1 [Reference]	1 [Reference]	1 [Reference]	1 [Reference]
Laparoscopic	0.90 (0.79-1.01)	0.83 (0.72-0.93)	0.80 (0.68-0.91)	NA	NA
Open	0.98 (0.86-1.12)	0.90 (0.79-1.02)	0.87 (0.74-0.99)	NA	NA
*ICD-10* (October 2015-2020)^b^					
Robotic-assisted	1 [Reference]	1 [Reference]	1 [Reference]	1 [Reference]	1 [Reference]
Laparoscopic	0.99 (0.88-1.11)	0.93 (0.80-1.05)	0.89 (0.75-1.03)	NA	NA
Open	0.89 (0.82-0.96)	0.81 (0.72-0.90)	0.78 (0.67-0.88)	NA	NA
**Individual surgeon robotic-assisted ventral hernia repair volume (percentile rank)**					
0%-25%					
Robotic-assisted	1 [Reference]	1 [Reference]	1 [Reference]	1 [Reference]	1 [Reference]
Laparoscopic	0.98 (0.74-1.24)	0.91 (0.69-1.13)	0.88 (0.66-1.09)	0.85 (0.64-1.07)	0.83 (0.62-1.05)
Open	0.99 (0.85-1.15)	0.97 (0.76-1.19)	0.94 (0.73-1.15)	0.91 (0.70-1.13)	0.89 (0.67-1.10)
26%-75%					
Robotic-assisted	1 [Reference]	1 [Reference]	1 [Reference]	1 [Reference]	1 [Reference]
Laparoscopic	0.87 (0.71-1.03)	0.80 (0.64-0.95)	0.77 (0.61-0.93)	0.75 (0.59-0.91)	0.73 (0.56-0.89)
Open	0.98 (0.88-1.12)	0.92 (0.77-1.07)	0.88 (0.72-1.05)	0.86 (0.69-1.03)	0.84 (0.66-1.01)
76%-100%					
Robotic-assisted	1 [Reference]	1 [Reference]	1 [Reference]	1 [Reference]	1 [Reference]
Laparoscopic	0.84 (0.73-0.96)	0.78 (0.66-0.89)	0.75 (0.63-0.87)	0.73 (0.60-0.85)	0.71 (0.58-0.84)
Open	0.99 (0.84-1.14)	0.92 (0.79-1.05)	0.88 (0.74-1.02)	0.86 (0.71-1.01)	0.84 (0.68-0.99)
**Individual surgeon robotic-assisted ventral hernia repair proportion of all ventral hernia repairs**					
0%-25%					
Robotic-assisted	1 [Reference]	1 [Reference]	1 [Reference]	1 [Reference]	1 [Reference]
Laparoscopic	0.87 (0.65-1.09)	0.79 (0.60-0.99)	0.76 (0.57-0.96)	0.74 (0.55-0.93)	0.72 (0.52-0.91)
Open	0.99 (0.78-1.20)	0.90 (0.70-1.11)	0.87 (0.66-1.07)	0.84 (0.63-1.05)	0.82 (0.60-1.03)
26%-75%					
Robotic-assisted	1 [Reference]	1 [Reference]	1 [Reference]	1 [Reference]	1 [Reference]
Laparoscopic	0.86 (0.73-0.99)	0.79 (0.66-0.91)	0.75 (0.63-0.88)	0.73 (0.60-0.86)	0.71 (0.57-0.85)
Open	0.97 (0.85-1.10)	0.89 (0.76-1.01)	0.85 (0.72-0.99)	0.83 (0.68-0.97)	0.80 (0.65-0.96)
76%-100%					
Robotic-assisted	1 [Reference]	1 [Reference]	1 [Reference]	1 [Reference]	1 [Reference]
Laparoscopic	0.93 (0.77-1.09)	0.85 (0.70-1.00)	0.81 (0.66-0.97)	0.79 (0.64-0.95)	0.77 (0.61-0.93)
Open	1.09 (0.93-1.24)	1.00 (0.84-1.15)	0.95 (0.79-1.12)	0.93 (0.76-1.10)	0.90 (0.72-1.08)

Abbreviations: *ICD-9*, *International Classification of Diseases, Ninth Revision*; *ICD-10*, *International Classification of Diseases, Tenth Edition*; NA, not applicable.

^a^ Myofascial flap analysis was performed using ventral or incisional subtype hernias only, as the technique is most commonly used for large or complex hernias and the number of umbilical hernias with myofascial flap composed less than 1% of all hernias coded with myofascial flap use.

^b^ Only 5 years of data were available for *ICD-9* and *ICD-10* sensitivity analyses.

## Discussion

In this analysis of Medicare beneficiaries from 2010 to 2020, the incidence of operative recurrence after ventral hernia repair was higher following robotic-assisted surgery compared with laparoscopic or open surgery. It is important to note that our findings should be interpreted within the context of evidence suggesting operative recurrence rates underestimate true clinical hernia recurrence by as much as 4 to 5 times.^28^ These results were consistent across different patient characteristics, hernia subtype (ventral/incisional or umbilical), and even among the highest-volume surgeons performing robotic-assisted ventral hernia repairs in the sample. These data suggest robotic-assisted ventral hernia repair has inferior long-term outcomes compared with established open and laparoscopic approaches, calling into question the clinical rationale behind its widespread and rapid adoption over the last decade.

The results of this study are complementary to and consistent with prior randomized clinical trials and observational studies. For example, a similar study from 2022 demonstrated higher rates of operative recurrence after minimally invasive vs open ventral hernia repair without differentiating between laparoscopic and robotic-assisted approaches.^21^ Our work expands on this finding by explicitly comparing operative recurrence between all 3 approaches. Our results conflict with earlier work in the same Medicare population from 2007 to 2015, which found fewer recurrences after robotic-assisted and laparoscopic repair compared to open repair.^20^ These differences are potentially due to our methods, as we used an IV analysis to address selection bias and other unmeasured sources of confounding inherent in observational data. Moreover, rates of robotic-assisted ventral hernia repair increased sharply starting in 2014, and our study captures the diffusion of this approach out of specialized centers and into the broader surgical community.

Current trends suggest that the use of robotic assistance will continue to grow, even without evidence of clinical superiority to other approaches. A 2022 study revealed that the major drivers of robotic-assisted hernia repair were not patient or hernia characteristics but rather market competition and availability of the robotic console.^29^ Moreover, proponents of the robotic platform argue that the advantages over laparoscopy for the surgeon, such as an easier learning curve and improved surgical ergonomics, may justify its use even in the absence of clinical benefit for patients.^4,5,6,7^ While evidence to support these proposed advantages remains mixed,^30,31,32^ our study highlights the potential downside for patients when demand for a new technology outpaces the availability of high-quality evidence.

One area where robotic assistance may offer a clinical advantage is in repairing large or complex hernias with techniques that could not otherwise be performed laparoscopically. For example, robotic-assisted component separation, a commonly used abdominal wall reconstructive technique (coded as myofascial flap in claims data), is associated with shorter length of stay and fewer complications compared to open repair.^33^ However, most surgeons across the country are not using robotic assistance for these complex repairs, which are typically performed by only the most experienced abdominal wall surgeons. In our study, higher surgeon robotic-assisted ventral hernia repair volume did lessen the magnitude of differences in operative recurrence rates, but did not eliminate differences entirely. Thus, experience may be a crucial component to improving outcomes with robotic-assisted hernia repair, and the ability to right-size adoption necessitates improved training and credentialing that align with the modern realities of surgical practice.

Increases in the incidence and complexity of abdominal wall hernias have led to a push for hernia-specific fellowship training.^34^ While this may improve the pipeline of surgeons with hernia expertise, it does not address the broader community of general surgeons who perform most hernia repairs in the US. Various institution-level robotic training curricula have been implemented in surgical residencies across the country, but the American Board of Surgery does not require standardized robotic training or assessment for surgical trainees. This has produced widely varying training components, case exposure, and documentation of competency on residency graduation.^35^ Furthermore, access to robotic simulators and adequate case volumes during training is largely institution dependent, producing additional barriers to personalized trainee competency assessment.^36^ For surgeons who desire robotic-assisted surgical training postresidency, credentialing requirements typically involve completion of an industry-sponsored training course and performance of a certain number of proctored robotic-assisted cases.^37^ A 2021 study found these credentialing processes inadequate to determine proficiency due to varying requirements, lack of alignment of case numbers with procedure-specific learning curves, and an absence of outcome monitoring.^38^ Standardized training and credentialing for robotic-assisted surgery would provide a foundation on which to better understand a surgeon’s proficiency and safety prior to using the technology in independent practice.

### Limitations

Our work must be understood in the context of several limitations. First, the Medicare population may not be generalizable to all patients with hernias. However, ventral hernia repair is more common in older adults due to both aging and the higher likelihood of developing a hernia from a prior abdominal operation.^39^ Second, Medicare claims data are not able to capture nuanced clinical information, such as hernia size or mesh type. However, as most surgeons are not abdominal wall specialists, we would expect Medicare claims to reflect an average general surgical practice, where most robotic-assisted hernia repairs are performed on patients with smaller or less complex ventral hernias. Third, selection bias is a known limitation of observational studies; our study was designed specifically to address this issue by using an IV analysis. We confirmed mathematically that this instrument was strong and conformed to the standards of exogeneity. Finally, it is possible that our study period captures the global learning curve that accompanies the use of a new technique, similar to the early studies that showed laparoscopic ventral hernia repairs had higher recurrence rates than open repairs.^40^ While the differences we found may diminish once robotic assistance is used more universally, the incremental value added by the robot would remain in question. Even in the presence of similar outcomes, laparoscopy would still have the advantage of lower costs and resource use compared with robotic-assisted ventral hernia repair.

## Conclusions

In this population-level observational study that used an IV analysis to control for unmeasured confounding, patients undergoing robotic-assisted ventral hernia repair had higher rates of operative recurrence than those undergoing laparoscopic or open repair up to 10 years after surgery. Narrower clinical applications that focus on the specific advantages and disadvantages of each approach may improve patient outcomes following ventral hernia repairs.

## Notes

Supplement 1. **eFigure 1**. Stepwise Patient Inclusion and Exclusion and Final Index Ventral Hernia Repair Cohort Study Size **eFigure 2**. Yearly Distribution of Elective, Inpatient Ventral, Incisional, and Umbilical Hernia Repairs From 2010-2020, by Approach **eMethods 1**. Race and Ethnicity Use in Medicare Claims Database **eMethods 2**. Selection of Instrumental Variable and Intuition for Instrumental Variable Analysis **eTable 1**. *ICD-9*, *ICD-10*, and *CPT* Codes Used to Identify Initial Hernia Repair Operation and Reoperation **eTable 2**. Covariate Balance Grouped by Actual Treatment and by Above and Below Median of Prior Year Robotic-Assisted Use Rate for Robotic-Assisted vs Laparoscopic Approaches **eTable 3. **Covariate Balance Grouped by Actual Treatment and by Above and Below Median of Prior Year Robotic-Assisted Use Rate for Robotic-Assisted vs Open Approaches
eReferences.

Supplement 2. Data Sharing Statement
