# Supplementary material for: Surgical Approach and Long-Term Recurrence After Ventral Hernia Repair
Source: JAMA Surg. 2024 Jun 12;159(9):1019–28. doi: 10.1001/jamasurg.2024.1696 (PMC11170458; doi:10.1001/jamasurg.2024.1696)
Supplement: Supplement 1. — eFigure 1. Stepwise Patient Inclusion and Exclusion and Final Index Ventral Hernia Repair Cohort Study Size eFigure 2. Yearly Distribution of Elective, Inpatient Ventral, Incisional, and Umbilical Hernia Repairs From 2010-2020, by Approach eMethods 1. Race and Ethnicity Use in Medicare Claims Database eMethods 2. Selection of Instrumental Variable and Intuition for Instrumental Variable Analysis eTable 1. ICD-9, ICD-10, and CPT Codes Used to Identify Initial Hernia Repair Operation and Reoperation eTable 2. Covariate Balance Grouped by Actual Treatment and by Above and Below Median of Prior Year Robotic-Assisted Use Rate for Robotic-Assisted vs Laparoscopic Approaches eTable 3. Covariate Balance Grouped by Actual Treatment and by Above and Below Median of Prior Year Robotic-Assisted Use Rate for Robotic-Assisted vs Open Approaches eReferences. [file jamasurg-e241696-s001.pdf]

Supplemental Online Content

Fry BT, Howard RA, Thumma JR, Norton EC, Dimick JB, Sheetz KH. Surgical approach and long-term recurrence after ventral hernia repair. *JAMA Surg*. Published online June 12, 2024. doi:10.1001/jamasurg.2024.1696

eFigure 1. Stepwise Patient Inclusion and Exclusion and Final Index Ventral Hernia Repair Cohort Study Size
eFigure 2. Yearly Distribution of Elective, Inpatient Ventral, Incisional, and Umbilical Hernia Repairs From 2010-2020, by Approach
eMethods 1. Race and Ethnicity Use in Medicare Claims Database
eMethods 2. Selection of Instrumental Variable and Intuition for Instrumental Variable Analysis
eTable 1. *ICD-9*, *ICD-10*, and *CPT* Codes Used to Identify Initial Hernia Repair Operation and Reoperation
eTable 2. Covariate Balance Grouped by Actual Treatment and by Above and Below Median of Prior Year Robotic-Assisted Use Rate for Robotic-Assisted vs Laparoscopic Approaches
eTable 3. Covariate Balance Grouped by Actual Treatment and by Above and Below Median of Prior Year Robotic-Assisted Use Rate for Robotic-Assisted vs Open Approaches
eReferences

This supplemental material has been provided by the authors to give readers additional information about their work.

© 2024 Fry BT et al. *JAMA Surgery.*

eFigure 1. Stepwise Patient Inclusion and Exclusion and Final Index Ventral Hernia Repair Cohort Study Size

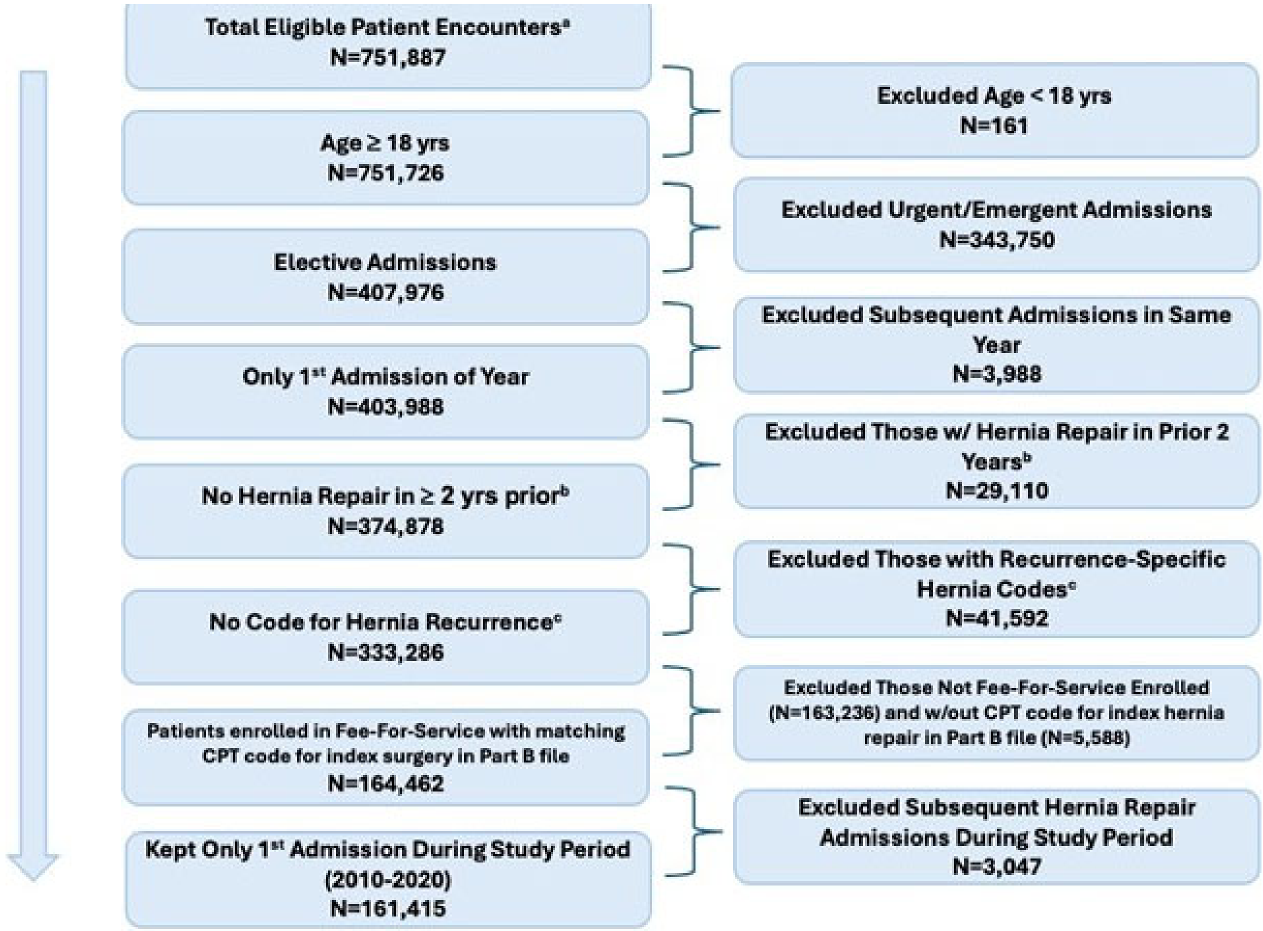

[a]Patients initially identified using International Classification of Diseases 9th and 10th Edition (ICD-9/10) procedure codes, which were cross referenced with the corresponding ICD-9/10 diagnosis codes (**eTable 1 in Supplement**)
[b]Because not all recurrent hernias repairs are coded as such, we reduced the possibility of mis-identifying index hernia repairs by excluding patients with an additional ventral hernia repair admission dating back to 2008, or at least the 2 years prior to their year of index surgery.
[c]Recurrent hernia specific Current Procedural Terminology (CPT) codes were 49565, 49566, 49656, 49657

© 2024 Fry BT et al. *JAMA Surgery*.

eFigure 2. Yearly Distribution of Elective, Inpatient Ventral, Incisional, and Umbilical Hernia Repairs From 2010-2020, by Approach

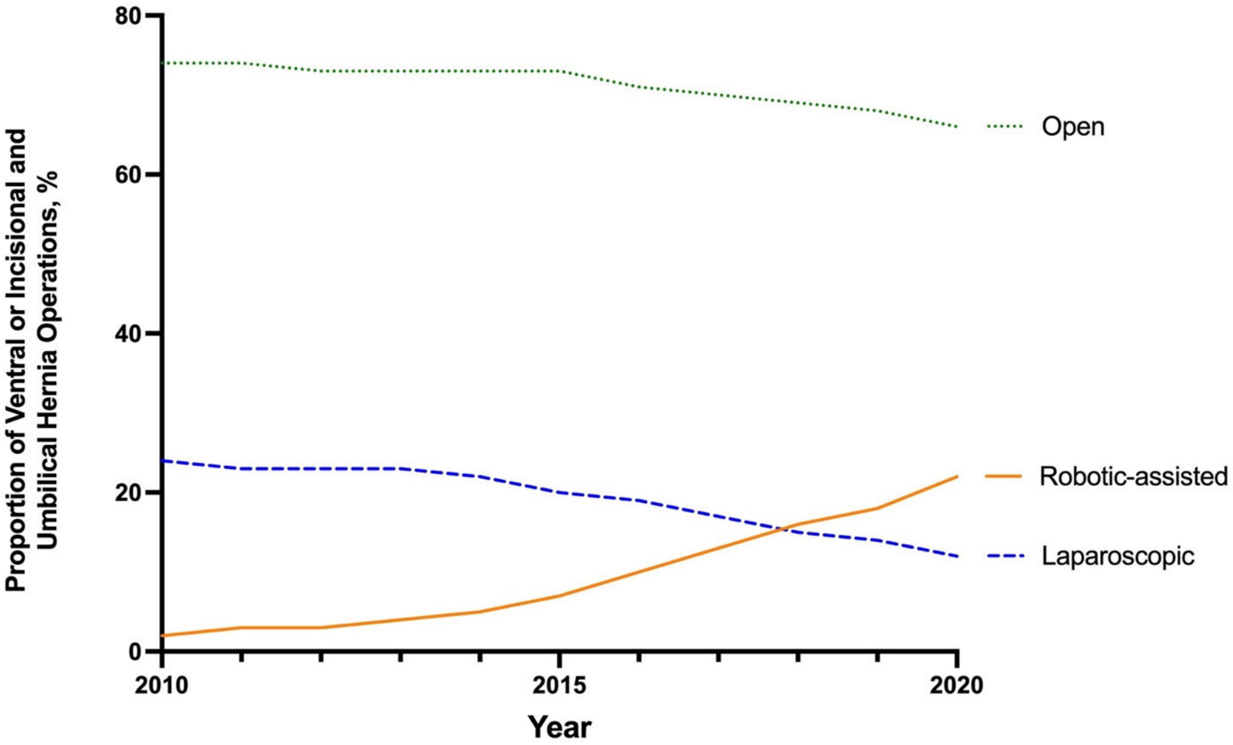

© 2024 Fry BT et al. *JAMA Surgery.*

eMethods 1. Race and Ethnicity Use in Medicare Claims Database

Patient race and ethnicity are included as identifiers in the Medicare claims database.[1] These identifiers were used to identify patient race and ethnicity in this study, and are represented in the following standard, fixed categories: Asian, Black, Hispanic, North American Native, White, other race/ethnicity, and unknown race/ethnicity. Only a single category could be selected during our study period, and no additional information is available in Medicare claims data regarding the composition of the “other race” category. Race and ethnicity were included as they have been found to be associated with the presentation, management, and outcomes of hernias and hernia repair.[2-4]

© 2024 Fry BT et al. *JAMA Surgery.*

eMethods 2. Selection of Instrumental Variable and Intuition for Instrumental Variable Analysis

As mentioned in the main text of the manuscript, due to measured and unmeasured confounding in observational data, causal inference may be limited (e.g., due to selection bias). For example, surgeons may choose an open approach for large complex hernias in sicker patients versus a minimally invasive approach (robotic-assisted or laparoscopic) for small simple hernias in healthy patients. Moreover, there are surgeons with robotic expertise who may use the technology to perform more complex hernia repairs that could not otherwise be performed in a minimally invasive fashion; conversely, surgeons uncomfortable with performing laparoscopic hernia repair may use the robot for simple, straightforward hernias to provide a minimally invasive alternative for an operation that would have otherwise been performed open. We cannot account for these biases with standard multivariable logistic regression analysis, as it can only adjust for measured covariates. To mitigate this, an instrumental variable (IV) analysis identifies robotic-assisted hernia repair patients who would have otherwise undergone an open or laparoscopic approach at a different institution. The IV is an exogenous factor associated with the likelihood of receiving a particular treatment without affecting the treatment outcome. In turn, this effectively randomizes treatment for a subset of patients which improves causal inference from observational data.

The instrumental variable used for our analysis was the robotic-assisted ventral hernia repair utilization rate within a hospital referral region in the 12 months prior to a patient's initial ventral hernia repair. The use of prior year state/regional level procedural utilization as an IV is consistent with previous observational studies in bariatric, colorectal, and vascular surgery patients.

© 2024 Fry BT et al. *JAMA Surgery.*

eTable 1. *ICD-9*, *ICD-10*, and *CPT* Codes Used to Identify Initial Hernia Repair Operation and Reoperation[a]

ICD-9 Diagnosis Codes	551.1, 551.2, 551.20, 551.21, 551.29, 552.1, 552.2, 552.20, 552.21, 552.29, 553.1, 553.2, 553.20, 553.21, 553.29
ICD-9 Procedure Codes	53.4, 53.41, 53.42, 53.43, 53.49, 53.5, 53.51, 53.59, 53.6, 53.61, 53.62, 53.63, 53.69
ICD-10 Diagnosis Codes	K42, K42.0, K42.1, K42.9, K43, K43.0, K43.1, K43.2, K43.6, K43.7, K43.9
ICD-10 Procedure Codes	0WQF0ZZ, 0WMF0ZZ, 0WQF0ZZ, 0WQF3ZZ 0WQF4ZZ, 0WQFXZZ, 0WUF07Z, 0WUF0JZ, 0WUF0KZ, 0WUF47Z, 0WUF4JZ, 0WUF4KZ
CPT Codes	49560, 49561, 49585, 49587, 49652, 49653, 49654, 49655, 49570, 49572
Robotic-assisted Codes	*ICD-9*: 1741, 1742, 1743, 1744, 1749<br>*ICD-10*: 8E0W0CZ, 8E0W3CZ, 8E0W4CZ, 8E0W4CZ, 8E0W7CZ, 8E0W8CZ, 8E0WXCZ<br>*CPT*: S2900

ICD-9: International Classification of Diseases 9 Edition; ICD-10: International Classification of Diseases 10th Edition; CPT: Current Procedural Terminology.
[a]Operative hernia recurrences were identified using the same codes, in addition to 4 additional CPT codes excluded from the initial operation that specifically identify recurrent hernia repair: 49565, 49566, 49656, 49657.

eTable 2. Covariate Balance Grouped by Actual Treatment and by Above and Below Median of Prior Year Robotic-Assisted Use Rate for Robotic-Assisted vs Laparoscopic Approaches[a,b]

	Actual Treatment				Prior Year Robotic-Assisted Ventral Hernia Repair Utilization Rate[c]			
	Robotic-Assisted (N=12,693)	Laparoscopic (N=32,542)	Standardized Difference (%)[d]	P-Value	Above median (N=80,943)	Below Median (N=80,472)	Standardized Difference (%)[d]	P-Value
Age, mean (SD)	69.9 (8.9)	68.5 (11.1)	14.4	<.0001	69.2 (10.5)	68.7 (11.2)	4.5	<.0001
Female	5810 (45.8)	19992 (61.4)	31.8	<.0001	46055 (56.9)	47768 (59.4)	5	<.0001
Race/Ethnicity								
White	10911 (86)	28285 (86.9)	2.8	0.007	69178 (85.5)	70001 (87)	4.4	<.0001
Black	1048 (8.3)	2707 (8.3)	0.2	0.83	6803 (8.4)	7082 (8.8)	1.4	0.005
Hispanic	227 (1.8)	593 (1.8)	0.3	0.808	1756 (2.2)	1399 (1.7)	3.1	<.0001
2+ Elixhauser Comorbidities	8337 (65.7)	22396 (68.8)	6.7	<.0001	57898 (71.5)	54694 (68)	7.8	<.0001
Hypertension	8640 (68.1)	22072 (67.8)	0.5	0.619	55172 (68.2)	53051 (65.9)	4.8	<.0001
Fluid/electrolyte disorders	1565 (12.3)	3571 (11)	4.2	<.0001	13099 (16.2)	11175 (13.9)	6.4	<.0001
Diabetes without chronic complications	2415 (19)	8077 (24.8)	14	<.0001	16548 (20.4)	20083 (25)	10.8	<.0001
Chronic pulmonary disease	2683 (21.1)	8014 (24.6)	8.3	<.0001	18999 (23.5)	18841 (23.4)	0.1	0.78
Obesity	3281 (25.8)	7606 (23.4)	5.8	<.0001	20899 (25.8)	17021 (21.2)	11	<.0001
Hypothyroidism	1861 (14.7)	5215 (16)	3.8	<.0001	13383 (16.5)	11608 (14.4)	5.8	<.0001
Anemias	1211 (9.5)	2915 (9)	2	0.053	9842 (12.2)	8497 (10.6)	5	<.0001
Renal failure	1405 (11.1)	3304 (10.2)	3	0.004	10513 (13)	8199 (10.2)	8.8	<.0001
Congestive heart failure	920 (7.2)	2372 (7.3)	0.2	0.88	6683 (8.3)	5972 (7.4)	3.1	<.0001
Depression	1400 (11)	4270 (13.1)	6.4	<.0001	10379 (12.8)	9137 (11.4)	4.5	<.0001
Diabetes with chronic complications	1076 (8.5)	1695 (5.2)	13	<.0001	6962 (8.6)	2824 (3.5)	21.5	<.0001
Liver disease	428 (3.4)	1563 (4.8)	7.2	<.0001	4249 (5.2)	2914 (3.6)	7.9	<.0001
Other neurological disorders	492 (3.9)	1523 (4.7)	4	<.0001	4078 (5)	3699 (4.6)	2.1	<.0001
Peripheral vascular disease	511 (4)	1577 (4.8)	4	<.0001	4193 (5.2)	4107 (5.1)	0.3	0.486
Valvular disease	556 (4.4)	1522 (4.7)	1.4	0.176	3935 (4.9)	3552 (4.4)	2.1	<.0001
Weight loss	263 (2.1)	535 (1.6)	3.2	0.002	2716 (3.4)	2439 (3)	1.8	<.0001
Coagulopathy	299 (2.4)	629 (1.9)	2.9	0.004	2365 (2.9)	1948 (2.4)	3.1	<.0001
Rheumatoid arthritis/ collagen vascular disease	354 (2.8)	1017 (3.1)	2	0.061	2692 (3.3)	2412 (3)	1.9	<.0001
Psychoses	262 (2.1)	907 (2.8)	4.7	<.0001	2280 (2.8)	2298 (2.9)	0.2	0.638
Solid tumor without metastasis	267 (2.1)	470 (1.4)	5	<.0001	1941 (2.4)	1425 (1.8)	4.4	<.0001
Metastatic cancer	300 (2.4)	344 (1.1)	10.1	<.0001	2297 (2.8)	1999 (2.5)	2.2	<.0001

[a]Median of the instrumental variable was 4.79% with a mean of 7.82% (SD 8.7%)
[b]All values represented as N (%) unless otherwise indicated. Only covariates with at least 2% prevalence in either approach reported.
[c]Prior year robotic-assisted ventral hernia repair utilization rate was the instrumental variable used in the analysis. This was evaluated at the hospital referral region level and calculated for the 12 months prior to each patient's initial hernia repair.
[d]Standardized difference equals mean difference divided by pooled standard deviation

© 2024 Fry BT et al. *JAMA Surgery.*

eTable 3. Covariate Balance Grouped by Actual Treatment and by Above and Below Median of Prior Year Robotic-Assisted Use Rate for Robotic-Assisted vs Open Approaches[a,b]

	Actual Treatment				Prior Year Robotic-Assisted Ventral Hernia Repair Utilization Rate[c]			
	Robotic-Assisted (N=12,693)	Open (N=116,180)	Standardized Difference (%)[d]	P-Value	Above median (N=80,943)	Below Median (N=80,472)	Standardized Difference (%)[d]	P-Value
Age, mean (SD)	69.9 (8.9)	69.0 (10.9)	9.1	<.0001	69.2 (10.5)	68.7 (11.2)	4.5	<.0001
Female	5810 (45.8)	68021 (58.5)	25.8	<.0001	46055 (56.9)	47768 (59.4)	5	<.0001
Race/Ethnicity								
White	10911 (86)	99983 (86.1)	0.3	0.762	69178 (85.5)	70001 (87)	4.4	<.0001
Black	1048 (8.3)	10130 (8.7)	1.7	0.079	6803 (8.4)	7082 (8.8)	1.4	0.005
Hispanic	227 (1.8)	2335 (2)	1.6	0.09	1756 (2.2)	1399 (1.7)	3.1	<.0001
2+ Elixhauser Comorbidities	8337 (65.7)	81859 (70.5)	10.3	<.0001	57898 (71.5)	54694 (68)	7.8	<.0001
Hypertension	8640 (68.1)	77511 (66.7)	2.9	0.002	55172 (68.2)	53051 (65.9)	4.8	<.0001
Fluid/electrolyte disorders	1565 (12.3)	19138 (16.5)	11.8	<.0001	13099 (16.2)	11175 (13.9)	6.4	<.0001
Diabetes without chronic complications	2415 (19)	26139 (22.5)	8.6	<.0001	16548 (20.4)	20083 (25)	10.8	<.0001
Chronic pulmonary disease	2683 (21.1)	27143 (23.4)	5.4	<.0001	18999 (23.5)	18841 (23.4)	0.1	0.78
Obesity	3281 (25.8)	27033 (23.3)	6	<.0001	20899 (25.8)	17021 (21.2)	11	<.0001
Hypothyroidism	1861 (14.7)	17915 (15.4)	2.1	0.024	13383 (16.5)	11608 (14.4)	5.8	<.0001
Anemias	1211 (9.5)	14213 (12.2)	8.7	<.0001	9842 (12.2)	8497 (10.6)	5	<.0001
Renal failure	1405 (11.1)	14003 (12.1)	3.1	0.001	10513 (13)	8199 (10.2)	8.8	<.0001
Congestive heart failure	920 (7.2)	9363 (8.1)	3.1	0.001	6683 (8.3)	5972 (7.4)	3.1	<.0001
Depression	1400 (11)	13846 (11.9)	2.8	0.003	10379 (12.8)	9137 (11.4)	4.5	<.0001
Diabetes with chronic complications	1076 (8.5)	7015 (6)	9.4	<.0001	6962 (8.6)	2824 (3.5)	21.5	<.0001
Liver disease	428 (3.4)	5172 (4.5)	5.6	<.0001	4249 (5.2)	2914 (3.6)	7.9	<.0001
Other neurological disorders	492 (3.9)	5762 (5)	5.3	<.0001	4078 (5)	3699 (4.6)	2.1	<.0001
Peripheral vascular disease	511 (4)	6212 (5.3)	6.3	<.0001	4193 (5.2)	4107 (5.1)	0.3	0.486
Valvular disease	556 (4.4)	5409 (4.7)	1.3	0.161	3935 (4.9)	3552 (4.4)	2.1	<.0001
Weight loss	263 (2.1)	4357 (3.8)	10	<.0001	2716 (3.4)	2439 (3)	1.8	<.0001
Coagulopathy	299 (2.4)	3385 (2.9)	3.5	<.0001	2365 (2.9)	1948 (2.4)	3.1	<.0001
Rheumatoid arthritis/ collagen vascular disease	354 (2.8)	3733 (3.2)	2.5	0.01	2692 (3.3)	2412 (3)	1.9	<.0001
Psychoses	262 (2.1)	3409 (2.9)	5.6	<.0001	2280 (2.8)	2298 (2.9)	0.2	0.638
Solid tumor without metastasis	267 (2.1)	2629 (2.3)	1.1	0.25	1941 (2.4)	1425 (1.8)	4.4	<.0001
Metastatic cancer	300 (2.4)	3652 (3.1)	4.8	<.0001	2297 (2.8)	1999 (2.5)	2.2	<.0001

[a]Median of the instrumental variable was 4.79% with a mean of 7.82% (SD 8.7%)
[b]All values represented as N (%) unless otherwise indicated. Only covariates with at least 2% prevalence in either approach reported.
[c]Prior year robotic-assisted ventral hernia repair utilization rate was the instrumental variable used in the analysis. This was evaluated at the hospital referral region level and calculated for the 12 months prior to each patient's initial hernia repair.
[d]Standardized difference equals mean difference divided by pooled standard deviation

© 2024 Fry BT et al. *JAMA Surgery.*

eReferences

1. Race/ethnicity (from Medicare EDB) | ResDAC. Resdac.org. Published 2022. Accessed January 13, 2022. https://resdac.org/cms-data/variables/raceethnicity-medicare-edb.
2. Bowman K, Telem DA, Hernandez-Rosa J, Stein N, Williams R, Divino CM. Impact of race and socioeconomic status on presentation and management of ventral hernias. *Arch Surg.* 2010;145(8):776-780.
3. Vu JV, Gunaseelan V, Dimick JB, Englesbe MJ, Campbell DA, Jr., Telem DA. Mechanisms of age and race differences in receiving minimally invasive inguinal hernia repair. *Surg Endosc.* 2019;33(12):4032-4037.
4. Asolati M, Huerta S, Sarosi G, Harmon R, Bell C, Anthony T. Predictors of recurrence in veteran patients with umbilical hernia: single center experience. *Am J Surg.* 2006;192(5):627-630.

© 2024 Fry BT et al. *JAMA Surgery.*
