# Supplementary material for: Surgical Approach and Long-Term Recurrence After Ventral Hernia Repair
Source: JAMA Surg. 2024 Jun 12;159(9):1019–28. doi: 10.1001/jamasurg.2024.1696 (PMC11170458; doi:10.1001/jamasurg.2024.1696)
Supplement: Supplement 2. — Data Sharing Statement [file jamasurg-e241696-s002.pdf]

Data Sharing Statement

Fry. Surgical Approach and Long-Term Recurrence After Ventral Hernia Repair. *JAMA Surg*. Published June 12, 2024. doi:10.1001/jamasurg.2024.1696

Data
Data available: No

Additional Information
Explanation for why data not available: We utilized Medicare claims data for this study and are unable to share the data based on our data usage agreement with Centers for Medicare & Medicaid Services.
